# Using an effective TB vaccination regimen to identify immune responses associated with protection in the murine model

**DOI:** 10.1016/j.vaccine.2021.01.034

**Published:** 2021-03-01

**Authors:** Nawamin Pinpathomrat, Naomi Bull, Janet Pasricha, Rachel Harrington-Kandt, Helen McShane, Elena Stylianou

**Affiliations:** a The Jenner Institute, University of Oxford, Old Road Campus Research Building, Roosevelt Drive, Oxford, United Kingdom

**Keywords:** Tuberculosis, Vaccine, Intranasal, Viral vector, BCG, Protection, Memory T cells, Immunogenicity

## Abstract

A vaccine against tuberculosis (TB), a disease resulting from infection with *Mycobacterium tuberculosis* (*M.tb*), is urgently needed to prevent more than a million deaths per year. Bacillus Calmette–Guérin (BCG) is the only available vaccine against TB but its efficacy varies throughout the world. Subunit vaccine candidates, based on recombinant viral vectors expressing mycobacterial antigens, are one of the strategies being developed to boost BCG-primed host immune responses and efficacy. A promising vaccination regimen composed of intradermal (i.d.) BCG prime, followed by intranasally (i.n.) administered chimpanzee adenoviral vector (ChAdOx1) and i.n. or i.d. modified vaccinia Ankara virus (MVA), both expressing Ag85A, has been previously reported to significantly improve BCG efficacy in mice. Effector and memory immune responses induced by BCG-ChAdOx1.85A-MVA85A (B-C-M), were evaluated to identify immune correlates of protection in mice. This protective regime induced strong Ag85A-specific cytokine responses in CD4^+^ and CD8^+^ T cells, both in the systemic and pulmonary compartments. Lung parenchymal CXCR3^+^ KLRG1^-^ Ag85A-specific memory CD4^+^ T cells were significantly increased in B-C-M compared to BCG immunised mice at 4, 8 and 20 weeks post vaccination, but the number of these cells decreased at the latter time point. This cell population was associated with the protective efficacy of this regime and may have an important protective role against *M.tb* infection.

## Highlights

• Boosting BCG with ChAdOx1.85A and MVA85A (B-C-M) improves its protective efficacy.
• B-C-M induces pulmonary and systemic Ag85A-specific cytokine and antibody responses.
• B-C-M enhances resident memory CD4^+^ and CD8^+^ T cells in the lung parenchyma.
• Protection associated with lung parenchymal Ag85A-specific CD4^+^ CXCR3^+^ KLRG1^-^ T cells.

## 1 Introduction

Tuberculosis (TB) is a global health issue, resulting from infection with *Mycobacterium tuberculosis* (*M.tb*). In 2018, the world health organization (WHO) reported 10 million new TB cases and 1.5 million deaths [1]. Bacillus Calmette–Guérin (BCG), first used in the 1920s, remains the only licensed vaccine for preventing TB [1]. BCG provides some protection against severe forms of paediatric TB but has highly variable protective efficacy against adult pulmonary TB [2]. There are no clear immunological biomarkers that correlate with protection, however we do have insight into some of the components of a protective immune response. It is widely acknowledged that CD4^+^ T cells are essential for protection against *M.tb.* Reduction of the CD4^+^ T cell count in HIV-infected patients results in an increased risk of developing active TB disease [3], [4], [5]. Interestingly, CD4^+^ T cells secreting *M.tb*-specific IFN-γ are intact in active TB patients [6]. There are no similar human associations suggesting a protective function of CD8^+^ T cells. However, it is clear that *M.tb* infection induces CD8^+^ T cells which can be cytokine producing and cytotoxic in humans [7], [8]. Furthermore, BCG-induced protection was abrogated in macaques in whom the CD8^+^ T cells had been depleted after infection with high dose *M.tb* [9]. In BCG vaccinated infants IFN-γ-secreting BCG-specific ELISPOT responses were associated with a reduced risk of TB disease [10].

The main aim of vaccination is the induction of memory T cells such as effector memory T cells (T_EM_) and resident memory T cells (T_RM_) [11]. In *M.tb* infection and BCG vaccination, T_EM_ (expressing high CD44 and low CD62L) are enhanced in murine lungs [12] and CD69 expression is crucial for their retainment in peripheral tissues [13]. In mice, lung CD4^+^ T_RM_ cells express CD11a, whereas CD8^+^ T_RM,_ express CD103 [14], [15]. Recently lung homing markers have been defined which allow discrimination between lung parenchymal and intravascular memory T cells [16]. The latter T cell population expresses high levels of CX3C-chemokine receptor 1 (CX3CR1) and killer cell lectin like receptor G1 (KLRG1) [17], [18]. The absence of KLRG1, together with the expression of CXC-chemokine receptor 3 (CXCR3) is used to phenotypically identify memory T cells in the lung parenchyma [17]. This population is enhanced after immunisation with a TB subunit vaccine, mucosal BCG or *M.tb* infection [17], [19], [20], [21].

The role of antibodies in TB disease remains unclear. It has been suggested that in some circumstances antibodies may do harm by causing immunological pathology in active disease [22]. To determine the function of anti-mycobacterial antibodies, serum therapy studies have been conducted in murine models, showing both protective and non-protective effects [23]. Recently, higher titres of Ag85A-specific IgG have been shown to be associated with reduced risk of developing active disease in infants [10]. Immunoglobulin G1 (IgG1) was able to enhance the production of TNF-α from monocytes in TB patients [24]. Immunoglobulin A (IgA) knockout mice had higher bacterial CFU numbers in the lungs compared to control mice after *M.tb* challenge [25]. Therefore antibodies may contribute to protection against *M.tb*, particularly when induced mucosally [26], [27]. For vaccine design, it is encouraging that mucosal vaccination can induce antibody responses systemically and locally which may improve protective immunity [28], [29]. More data is needed on the humoral response to vaccination and its contribution to protection.

Subunit vaccines are designed to boost BCG and to express one or more mycobacterial antigens. A Modified Vaccinia virus Ankara (MVA) expressing the Ag85A antigen from *M.tb* (MVA85A), was the first prophylactic TB vaccine candidate to be evaluated in a phase 2b efficacy trial but it was unable to improve the efficacy of BCG in infants [30], [31], [32]. Consequently, a novel replication-deficient chimpanzee adenovirus viral vector was developed to express the same antigen (ChAdOx1.85A), to evaluate whether heterologous prime boost regimens were more protective than MVA85A alone in preclinical models [33]. In mice, BCG-ChAdOx1.85A-MVA85A (B-C-M) regimen has consistently demonstrated superior protection compared to BCG alone when mice were challenged 4 weeks post vaccination [34]. Here, we analysed the durability of this protection at 8 and 20 weeks post vaccination. We show that B-C-M significantly improved BCG efficacy against *M.tb* infection at 8 weeks but it was only partially protective at 20 weeks post vaccination. The immunogenicity of B-C-M was then analysed to identify immune responses that associated with protection. The intravascular staining technique was used in combination with Ag85A-specific tetramers and surface staining of lung-homing markers to discriminate between lung vascular and parenchymal T cells. We found that vaccination with B-C-M significantly increased antigen-specific CXCR3^+^ KLRG1^-^ CD4^+^ T cells in the lung parenchyma compared to BCG alone suggesting that this cell population was associated with protection.

## 2 Results

### 2.1 Evaluation of the durability of protection provided by the B-C-M regimen

**Fig. 1:**
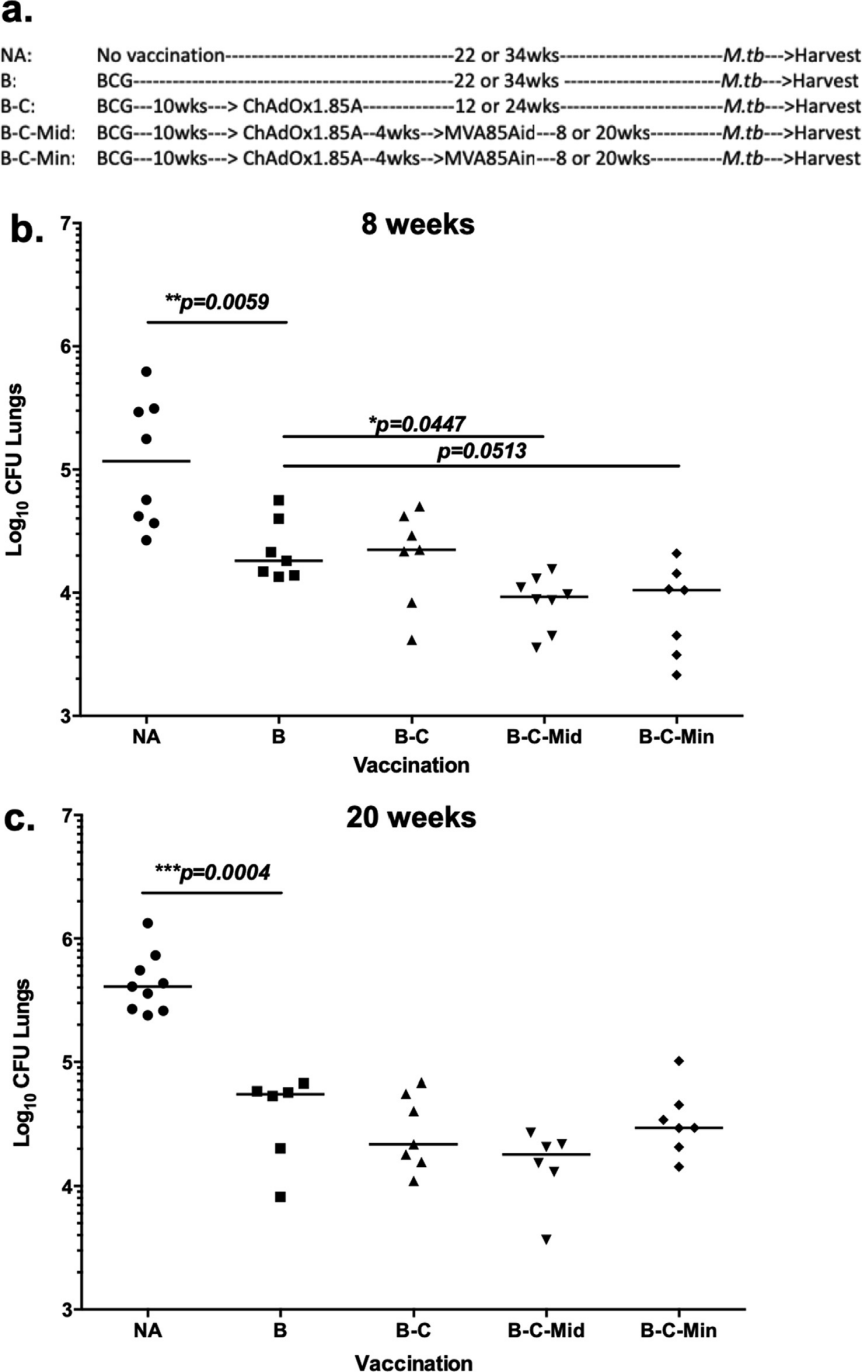
Assessment of the protective efficacy of the B-C-M regimen, 8 and 20 weeks post vaccination. (a) Experimental schema. (b) Eight or (c) 20 weeks after vaccination, animals were challenged with 50–100 CFU of aerosol *M.tb* Erdman. Four weeks post infection, lungs were harvested for CFU enumeration. Each symbol represents one animal and the line is the median of each group (n = 6–8 mice). Statistical significance was determined using Kruskal-Wallis test with Dunn's multiple comparisons test. *, p ≤ 0.05; **, p ≤ 0.01; ***, p ≤ 0.001.

The B-C-M vaccination regimen was shown to provide superior protection to BCG, 4 weeks post vaccination [34]. To investigate the durability of this protection, mice were vaccinated and challenged as shown in Fig. 1a.

At 8 weeks post vaccination, BCG vaccinated mice had significantly lower lung CFU compared to unvaccinated mice (p = 0.0059) (Fig. 1b). Boosting with ChAdOx1.85A (B-C) failed to further improve protection. A further boost with i.d. MVA85A (B-C-Mid) resulted in significant improvement compared to the BCG control group (p = 0.0447) (Fig. 1b). Boosting B-C with i.n. MVA85A, (B-C-Min) improved efficacy compared to the BCG group, but this comparison did not reach statistical significance (p = 0.0513) (Fig. 1b). Twenty weeks post immunisation, BCG was still protective (p = 0.0004) (Fig. 1c) and although B-C, B-C-Mid and B-C-Min all provided a small degree of improvement, none of these regimens was significantly better than BCG (Fig. 1c).

### 2.2 Antigen-specific antibody responses in the circulation and airways

**Fig. 2:**
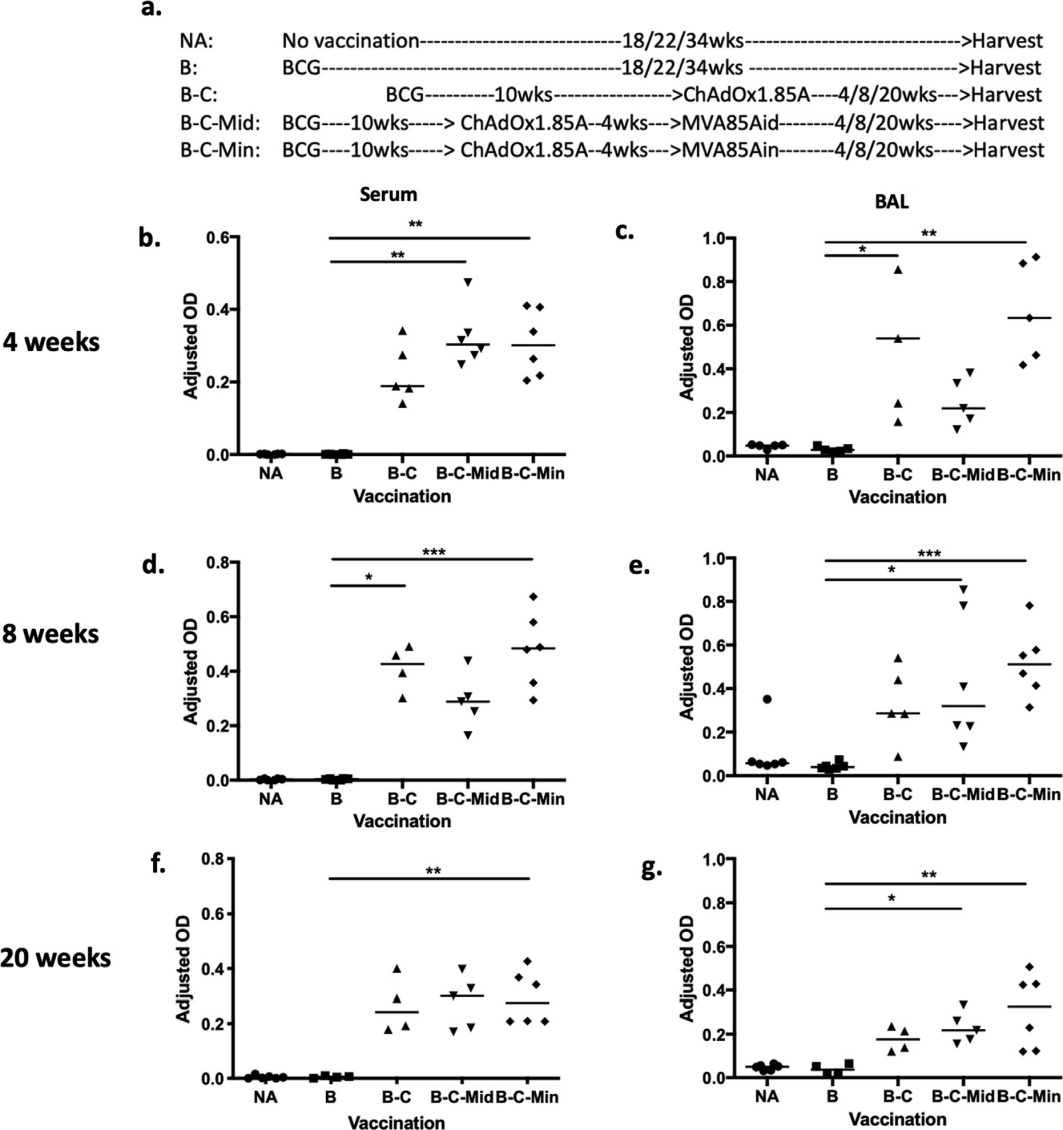
Ag85A-specific antibody responses in the circulation and airways of immunised and control mice at 4, 8 and 20 weeks post vaccination**.** Mice were vaccinated as shown in Fig. 2a and the samples were collected at (b-c) 4 weeks, (d-e) 8 weeks and (f-g) 20 weeks after vaccination completed. (b, d and f) Serum and (c, e and g) BAL samples were analysed using ELISA specific to Ag85A. (b) Total IgG in serum samples at 1:1350 dilution and (c) IgA in undiluted BAL samples were measured at 4 weeks post vaccination. At 8 and 20 week-time points, (d and f) total IgG and (e and g) IgA were presented respectively. Each symbol represents one animal and the line is the median of each group (n = 4–6 mice). Data are representative of two independent experiments. Statistical significance was determined using Kruskal-Wallis test with Dunn's multiple comparisons test. *, p ≤ 0.05; **, p ≤ 0.01; ***, p ≤ 0.001.

To investigate whether antibody responses correlate with vaccine-induced protection, serum and BAL samples were collected 4 weeks post vaccination. There were no detectable Ag85A-specific IgG in the serum of BCG vaccinated mice compared to unvaccinated controls (Fig. 2b). All booster vaccination regimens were able to increase serum Ag85A-IgG responses, with B-C-Mid and B-C-Min vaccinated mice reaching significance compared to BCG (p = 0.0023 and 0.0042 respectively) (Fig. 2b).

PPD-specific IgG was detected in the serum of all BCG vaccinated groups although the levels were not significant compared to the unvaccinated group (Supplement 1a). Boosting BCG with the viral vector vaccines did not enhance these responses (Supplement 1a).

There was no detectable Ag85A specific-IgA in the BAL fluid of the BCG group (Fig. 2c). However, a significant increase was observed in B-C and B-C-Min, compared to BCG vaccinated mice (p = 0.0117 and 0.0015 respectively), but not in B-C-Mid (Fig. 2c). Similarly, PPD-specific IgA responses increased after intranasal ChAdOx1.85A boost, but only reached significance in the B-C-Min, compared to the BCG group (p = 0.0004) (Supplement 1b).

Antigen-specific IgG was also measured at 8 and 20 weeks post vaccination. There were no detectable Ag85A-specific IgG responses in the serum of BCG group at either of the later time-points (Fig. 2d and f). However, boosting with ChAdOx1.85A (B-C) and with i.n. MVA85A (B-C-Min) significantly increased Ag85A-IgG compared to BCG at both time points (p = 0.0123, p = 0.0009 respectively) (Fig. 2d and f). Ag85A-specific IgG levels were still maintained 20 weeks post immunization, with only B-C-Min reaching significance compared to the BCG control (p = 0.0085) (Fig. 2f).

Airway Ag85A-specific IgA responses were also analysed at 8 and 20 weeks post vaccination. There were no detectable antibodies in the BCG group at either time points. All BCG boosted groups had an increase in antigen-specific IgA levels compared to the BCG group (B-C ns, B-C-Mid p = 0.0193, B-C-Min p = 0.0009) (Fig. 2e). The levels of Ag85A-specific IgA at the 20 weeks followed the same pattern with the result at 8 weeks showing a significant increase in IgA production in airways of B-C-Mid and B-C-Min vaccinated mice compared to BCG group (B-C ns, B-C-Mid p = 0.0262, B-C-Min p = 0.01) (Fig. 2g).

### 2.3 Resident memory T cells are induced by a protective vaccine regimen at 4 weeks post vaccination

**Fig. 3:**
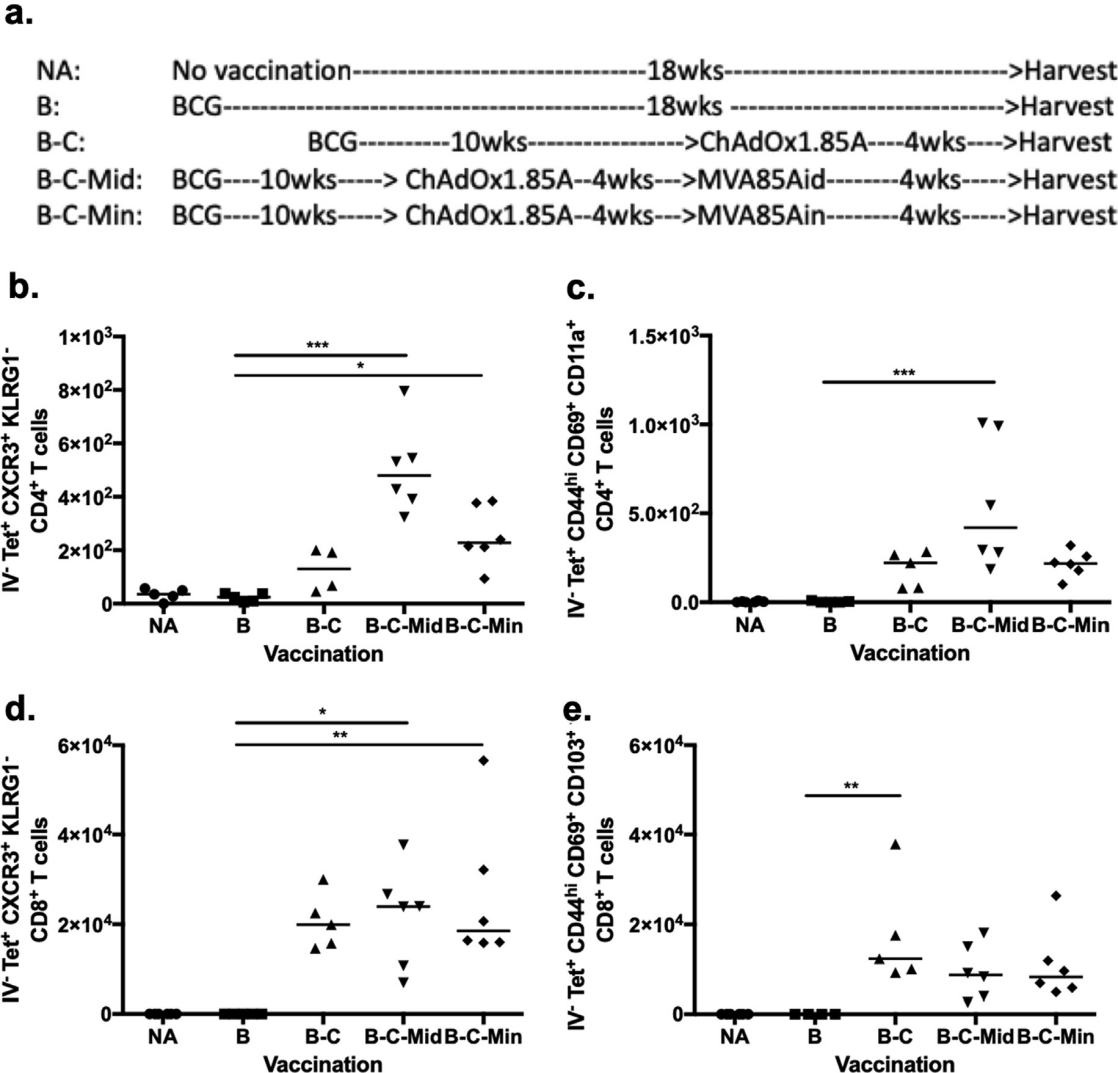
Characterization of the phenotype of antigen-specific T cells in the lung parenchyma of immunised mice and controls at 4 weeks post vaccination. (a) Experimental schema. Mice were vaccinated intradermally (i.d.) with 4x10^5^ CFU of BCG Pasteur (B) and boosted 10 weeks later with 1x10^8^ ifu of ChAdOx1.85A (C) intranasally (i.n.). Four weeks after ChAdOx1.85A vaccination, MVA85A was delivered either via the intradermal (Mid) or intranasal (Min) route in a dose of 5x10^6^ pfu. Four weeks post vaccination, CD4^+^ T cells (b and c) (Appendix 1) and CD8^+^ T cells (d and e) (Appendix 2) were analysed. (b) Ag85A-specific lung parenchymal CD4^+^ T cells expressing CXCR3^+^ KLRG1^-^ or (c) CD44^hi^ CD69^+^ CD11a^+^. (d) Ag85A-specific lung parenchymal CD8^+^ T cells expressing CXCR3^+^ KLRG1^-^ or (e) CD44^hi^ CD69^+^ CD103^+^. Each symbol represents one animal and the line is the median of each group (n = 4–6 mice). Data are representative of three independent experiments. Statistical significance was determined using Kruskal-Wallis test with Dunn's multiple comparisons test. *, p ≤ 0.05; **, p ≤ 0.01; ***, p ≤ 0.001.

To investigate antigen-specific CD4^+^ and CD8^+^ T cells induced by B-C-M, mice were vaccinated as per Fig. 3a. Intravascular staining was performed and lung samples were collected. Tetramer and surface staining were performed to classify memory CD4^+^ and CD8^+^ T cells at 4, 8 and 20 weeks post immunisation. Lung homing markers were used to identify lung parenchymal and intravascular memory T cells [35].

In the parenchyma, BCG did not induce significant levels of Ag85A-specific CD4^+^ T cells compared to the naïve control group (Fig. 3b and c). Four weeks after boosting BCG with intranasal ChAdOx1.85A (B-C) vaccination, Ag85A-specific CXCR3^+^ KLRG1^-^ CD4^+^ T cells were detected. A further boost with intradermal or intranasal MVA85A significantly increased this cell population (B-C-M). Both B-C-Mid and B-C-Min groups had a significantly higher number of cells compared to the BCG control group (p = 0.0002 and p = 0.0344 respectively) (Fig. 3b).

Resident memory T cell markers (CD44, CD62L, CD69 and CD11a) were also analysed to characterise the T_RM_ population, previously associated with protection against viral infection [14], [15]. Ag85A-specific lung parenchymal CD4^+^ T cells expressing CD44^hi^ CD62L^low^ CD69^+^ CD11a^+^ were significantly increased in B-C-Mid mice compared to BCG animals (p = 0.0005) (Fig. 3c),

Lung parenchymal CD8^+^ T cell responses were also investigated (Fig. 3d and e). BCG did not induce any detectable Ag85A-specific CD8^+^ CXCR3^+^ KLRG1^-^ T cells (Fig. 3d and e) but were increased after intranasal ChAdOx1.85A (p = 0.0511) (Fig. 3d). A further boost with MVA85A did not enhance this response further (Fig. 3d). In mice, CD4^+^ T_RM_ cells express CD11a, whereas CD8^+^ T_RM_ present CD103 in the lungs [14], [15]. Boosting BCG with intranasal ChAdOx1.85A resulted in a significant increase in Ag85A-specific lung parenchymal CD44^hi^ CD62L^low^ CD69^+^ CD103^+^ CD8^+^ T cells compared to BCG alone (p = 0.0029) (Fig. 3e). Boosting with MVA85A did not enhance this cell population further (Fig. 3e).

Boosting BCG with i.d. MVA85A alone, without a ChAdOx1.85A immunization (B-Mid), resulted in very low number of Ag85A-specific CD4 T_RM_ cells in the lungs of B-Mid vaccinated mice. These responses were lower than seen in the B-C, B-C-Mid and B-C-Min groups. (Supplement 4a). No Ag85A-specific CD8^+^ TRM responses were observed in the B-Mid vaccinated mice at 4 weeks after vaccination (Supplement 4b).

### 2.4 Longevity of resident memory T cells in lung parenchyma post B-C-M vaccination

**Fig. 4:**
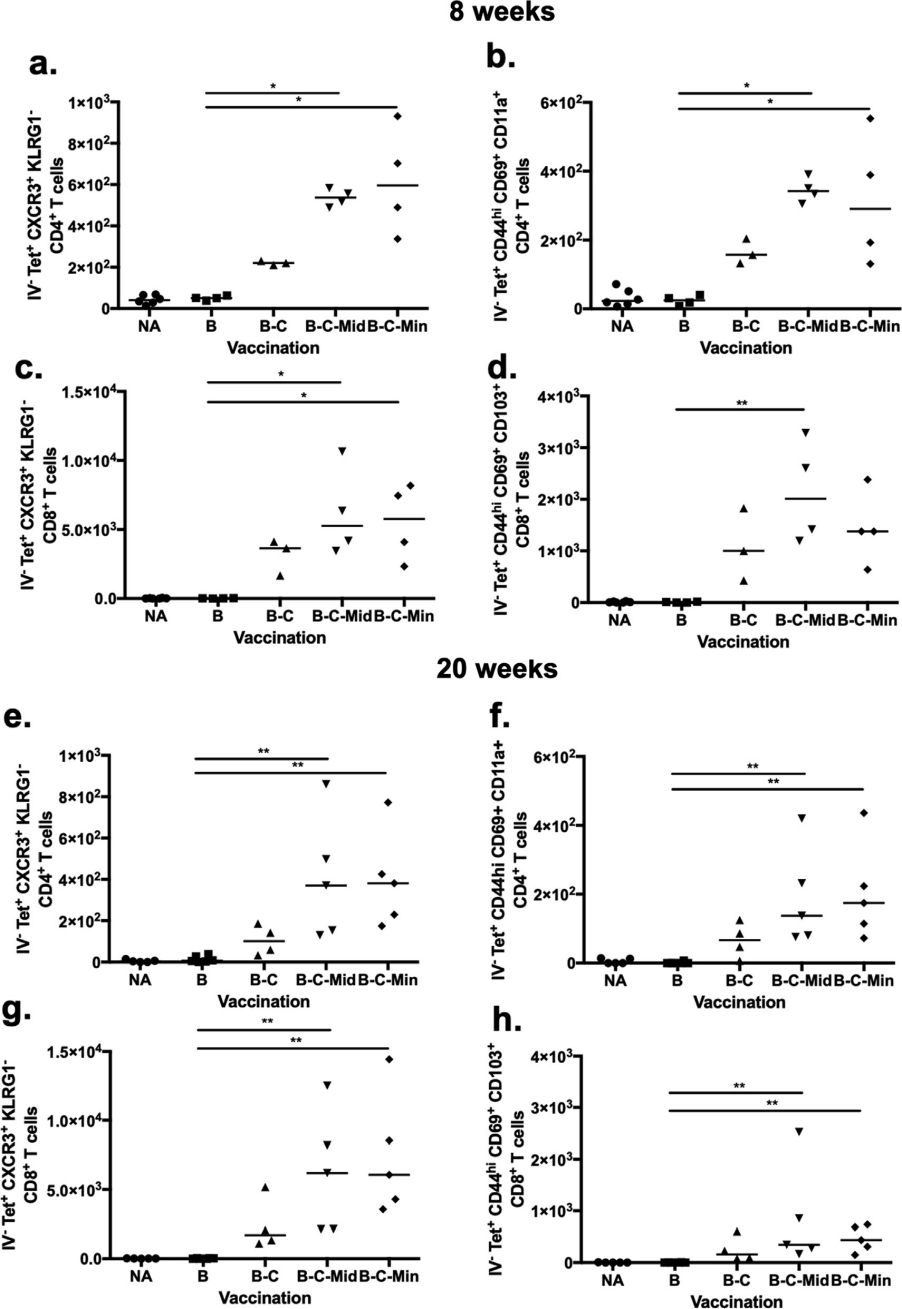
Characterization of the phenotype of antigen-specific T cells in the lung parenchyma of immunised mice and controls at 8 and 20 weeks post vaccination. Mice were vaccinated as shown in Fig. 2a. Lung cells were assessed 8 weeks post vaccination using flow cytometry and presented as cell number (1, 2). (a) Ag85A-specific lung parenchymal CXCR3^+^ KLRG1^-^ CD4^+^ T cells and (b) Ag85A-specific lung parenchymal CD44^hi^ CD69^+^ CD11a^+^ CD4^+^ T cells at 8 weeks after vaccination. (c) Ag85A-specific lung parenchymal CD8^+^ T cells expressed CXCR3^+^ KLRG1^-^. (d) Ag85A-specific lung parenchymal CD8^+^ T cells expressed CD44^hi^ CD69^+^ CD103^+^. A duplicate experiment was analysed at 20 week-time point. Lung parenchymal CD4^+^ and CD8^+^ T cells were phenotypically classified using the memory makers and presented in the same order (e-h). Each symbol represents one animal and the line is the median of each group (n = 3–6 mice). Data are representative of three independent experiments. Statistical significance was determined using Kruskal-Wallis test with Dunn's multiple comparisons test. *, p ≤ 0.05; **, p ≤ 0.01.

We also analysed lung homing and resident memory markers at 8 and 20 weeks post vaccination. Both markers showed an almost identical pattern at both time points (Fig. 4a-h). Ag85A-specific CXCR3^+^ and KLRG1^-^ CD4^+^ T cells were significantly increased in the lung parenchyma of B-C-Mid and B-C-Min compared to BCG immunised mice at 8 weeks (p = 0.0170 and p = 0.0103 respectively; Fig. 4a) and at 20 weeks (p = 0.0068 and p = 0.0026 respectively; Fig. 4e). At 8 weeks, the lung parenchymal CD4^+^ T cells expressing CD44^hi^ CD69^+^ CD11a^+^ were significantly enhanced in the B-C-Mid and B-C-Min groups compared to the BCG control mice (p = 0.0103 and p = 0.0428 respectively; Fig. 4b) and also at 20 weeks (p = 0.0063 and p = 0.0043 respectively; Fig. 4f). These two cell phenotypes were also increased in the lung parenchyma of B-C vaccinated mice but the differences were not statistically significant compared to BCG alone (Fig. 4a,b,e and f).

Lung homing and resident memory phenotype of CD8^+^ T cells were enhanced after BCG was boosted with ChAdOx1.85A compared to BCG alone but the difference did not reach statistical significance (Fig. 4c,d,g and h). After 8 and 20 weeks of the MVA85A boost, Ag85A-specific lung parenchymal CXCR3^+^, KLRG1^-^, CD8^+^ T cells were significantly increased in the lungs of B-C-Mid and B-C-Min compared to BCG immunised mice (p = 0.0170 and p = 0.0342 respectively; Fig. 4c, p = 0.0058 and p = 0.0022 respectively; Fig. 4g). Resident memory markers were also significantly increased in B-C-Mid group compared to BCG alone group (p = 0.0079) but not in B-C-Min group (p = 0.0802) (Fig. 4d) 8 weeks after immunisation. At the 20 week-time point, this T_RM_ population was significantly expanded in both groups compared to the BCG group (p = 0.0038 and p = 0.0068 respectively) (Fig. 4h). Overall, an MVA85A boost did not significantly improve B-C in inducing lung homing and resident memory phenotypes (Fig. 4).

### 2.5 Antigen-specific cytokine production of lung parenchymal T cells

Lung parenchymal CD4^+^ and CD8^+^ T cells expressing either lung-homing or resident memory markers were enhanced by the protective regimen, suggesting that these cell phenotypes might associate with protection. To determine the effector function of these cells, their ability to secrete Th1 cytokines was measured. Lung cells were stimulated *ex vivo* with PPD and Ag85A. PPD responses were evaluated to measure BCG induced/primed responses whereas Ag85A is to measure viral vector boost specific responses.

**Fig. 5:**
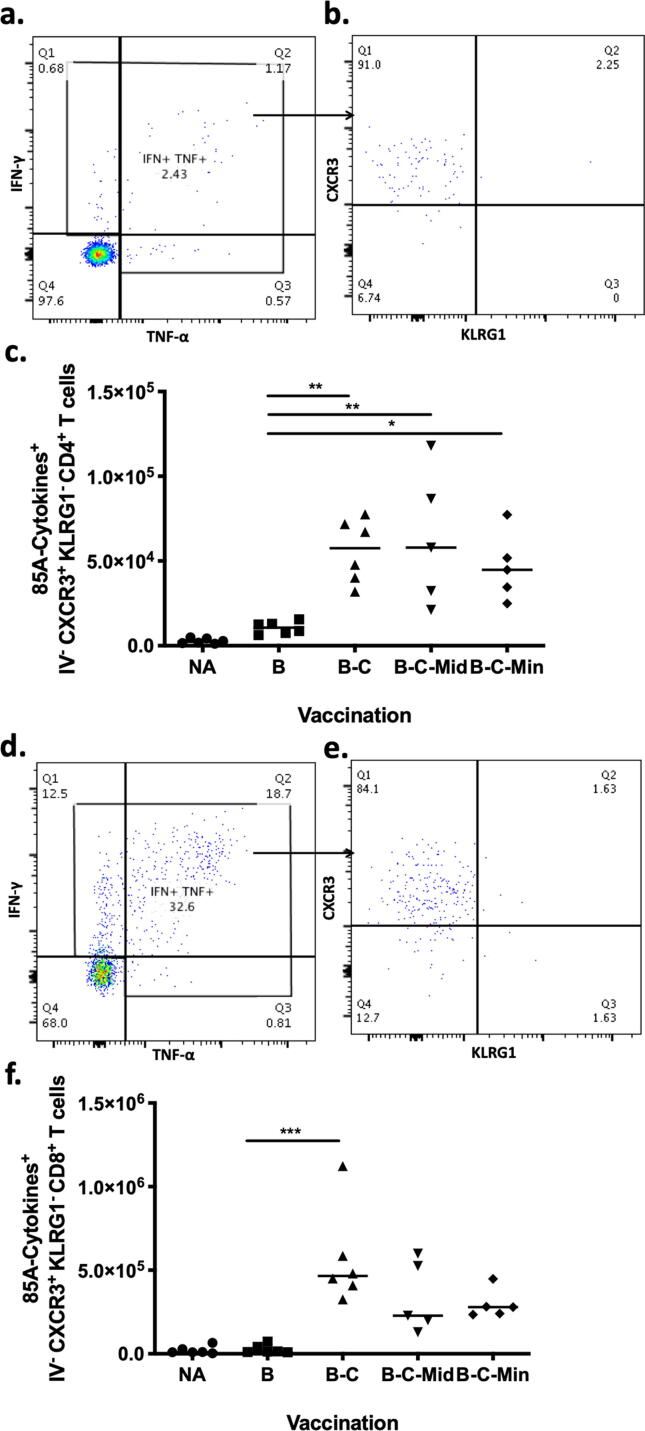
Assessing effector cytokine production of Ag85A-specific CD4^+^ T cells expressing CXCR3^+^ KLRG1^-^ in the lung parenchyma of naïve and vaccinated mice. Mice were immunised as shown in Fig. 3a and sacrificed 4 weeks post vaccination. Fluorochrome-conjugated CD45.2 was injected intravenously before sacrificing. Lungs cells were stimulated ex-vivo with Ag85A (85A) peptide pool. The cells were stained and analysed using flow cytometry (Appendix 3). (a) Representative flow plot shows lung parenchymal CD4^+^ T cells producing Ag85A-specific IFN-γ and/or TNF-α in the lungs of B-C-Mid vaccinated mice. (b) The cytokine producing cells were then gated on the expression of CXCR3^+^ KLRG1^-^. (c) Total cell number of Ag85A-specific IFN-γ and/or TNF-α producing lung parenchymal CXCR3^+^ KLRG1^-^ CD4^+^ T cells. (d) Representative flow plot shows lung parenchymal CD8^+^ T cells producing Ag85A-specific IFN-γ and/or TNF-α of B-C-Mid group. (e) The cytokine producing cells were then selected on the expression of CXCR3^+^ KLRG1^-^. (f) Total cell number of Ag85A-specific IFN-γ and/or TNF-α producing lung parenchymal CXCR3^+^ KLRG1^-^ CD8^+^ T cells. Each symbol represents one animal and the line is the median of each group (n = 4–6 mice). Data are representative of three independent experiments. Statistical significance was determined using Kruskal-Wallis test with Dunn's multiple comparisons test. *, p ≤ 0.05; **, p ≤ 0.01; ***, p ≤ 0.001.

Lung parenchymal CD4^+^ T cells were intracellularly stained to measure IFN-γ and TNF-α responses (Fig. 5a). The cytokine positive cells were then analysed based on the expression of CXCR3^+^ KLRG1^-^ (Fig. 5b). Very low levels of Ag85A-specific cytokine producing CD4^+^ T cells were detected in the BCG group. These cells were significantly enhanced with all boosting regimes compared to the BCG control group (B-C p = 0.0065, B-C-Mid p = 0.004 and B-C-Min p = 0.0409) (Fig. 5c). 90% of cytokine-secreting CD4^+^ T cells, in the lung parenchyma of B-C-Mid, were CXCR3^+^ KLRG1^-^ (Fig. 5b). IFN-γ and TNF-α responses were also observed in lung parenchymal CD8^+^ T cells (Fig. 5d). Lung homing markers were then selected from these cytokine-producing cells (Fig. 5e). No Ag85A-specific cytokine producing CD8^+^ T cells were detected in BCG group. Cytokine producing cells were significantly increased in the lung parenchyma of B-C vaccinated mice compared to BCG mice (p = 0.0005) but not in B-C-Mid and B-C-Min (Fig. 5f). In B-C-Mid vaccinated group, the majority of the cytokine producing CD8^+^ T cells (84%) expressed CXCR3^+^ KLRG1^-^ phenotype (Fig. 5e)

**Fig. 6:**
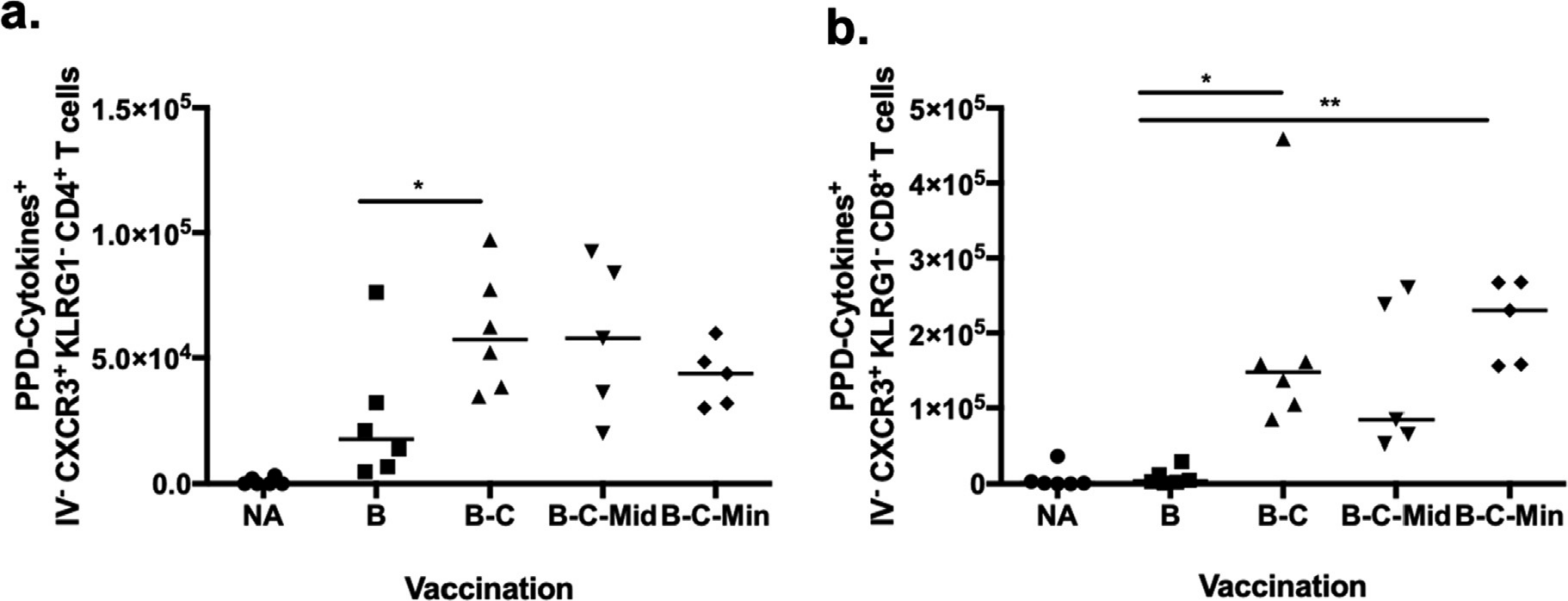
PPD-specific effector cytokine producing parenchymal CD4^+^ T cells and CD8^+^ T cells in the lungs of naïve and vaccinated mice at 4 weeks post vaccination. Mice were vaccinated as shown in Fig. 3a and sacrificed at 4 weeks post vaccination. Fluorochrome-conjugated CD45.2 was injected intravenously before sacrificing. Lungs cells were stimulated ex-vivo with PPD-T (PPD). The stimulated cells were analysed using flow cytometry (Appendix 3). Total cell number of PPD-specific IFN-γ and/or TNF-α producing lung parenchymal CXCR3^+^ KLRG1^-^ CD4^+^ T cells (a) and CD8^+^ T cells (b) in naïve and vaccinated mice. Each symbol represents one animal and the line is the median of each group (n = 4–6 mice). Data are representative of two independent experiments. Statistical significance was determined using Kruskal-Wallis test with Dunn's multiple comparisons test. *p ≤ 0.05; **, p ≤ 0.01.

PPD-specific cytokine producing CXCR3^+^ KLRG1^-^ CD4^+^ T cells were detected in the lungs of BCG vaccinated mice and were significantly increased in B-C mice (p = 0.0434) (Fig. 6a). A further boost with MVA did not enhance this cell population further (Fig. 6a). PPD-specific cytokine producing CXCR3^+^ KLRG1^-^ CD8^+^ T cells were significantly increased in the lung parenchyma of B-C and B-C-Min mice compared to BCG-vaccinated mice (p = 0.0116 and p = 0.0018) Fig. 6b). The responses induced by B-C-Mid were comparable with B-C and B-C-Min vaccination but were not significantly higher than the BCG controls (p = 0.0808) (Fig. 6b).

## 3 Discussion

Finding a definitive immune correlate of protection would be a significant tool in the development of an effective TB vaccine. Such a correlate would help to predict vaccine efficacy against *M.tb* infection and TB disease, saving time and costs involved with vaccine research and development. Here we used a protective heterologous BCG – ChAdOx1.85A – MVA85A prime-boost regimen in the murine model to identify potential immune correlates of protection.

The B-C-M regimen expressing Ag85A has been previously shown to be protective 4 weeks after vaccination [34]. To investigate the longevity of this protection, efficacy was investigated at 8 and 20 weeks post immunisation. Protection provided by B-C-M was significantly better to BCG at 8 weeks post vaccination. However, when mice were challenged 20 weeks post vaccination, although there was a trend for lower bacterial load, the difference was not significantly compared to BCG. It is likely that the protective effect of this regimen had waned at this time, as there was still a clear protective effect of BCG. It is also possible that differences in the *M.tb* infection dose between experiments could have affected the level of vaccine effect observed. Protection in preclinical animal experiments is always relative to challenge dose, and with small numbers of animals in each group, any minor amendments in infection levels could result in losses of significance.

At 4, 8 and 20 weeks post B-C-M vaccination, Ag85A-specific CD4^+^ T cells with a CXCR3^+^ KLRG1^-^ phenotype were increased in the lung-parenchyma, but the number of these cells was lower at the later time point. In our study, we have observed that lung parenchymal cells displaying a CXCR3^+^ KLRG1^-^ phenotype had a similar pattern to Ag85A-specific CD4^+^ T cells expressing CD44^hi^ CD69^+^ CD11a^+^. Delivery of BCG has also been shown to induce resident memory CD4^+^ T cells expressing CD44^hi^ CD69^+^ [36] and CXCR3^+^ KLRG1^-^ [20]. This lung homing population could be found in lungs of *M.tb* infected mice and was shown to confer protection against infection [17], [20]. In *M.tb* infection, the lung parenchymal CD4^+^ T cells expressing the lung homing phenotype were more protective compared to the intravascular population using adoptive transfer [17]. Circulating CXCR3^+^ CD4^+^ T cells were enhanced in the lung vasculature after intramuscular vaccination of an adenoviral-based vaccine however this was not a protective route [37]. In contrast, mucosal delivery of the same virus was protective against *M.tb* challenge [38]. Subcutaneous vaccination of another viral vector vaccine, resulted in the significant increase of cells with CXCR3^+^ and KLRG1^-^ phenotype in the circulation of vaccinated mice compared to unvaccinated controls [19]. This suggests that the lung homing CD4^+^ T cells were compartmentalised specifically to the route of vaccination or infection. These cells could provide protection when they were located at the lung parenchyma, not in the lung vasculature in the murine model. However, this is not necessarily the same in humans or NHP. In fact, the route of vaccination of AdHu5.85A appears less important for protective efficacy in NHPs [39]. Interestingly, intradermal vaccination with BCG provided consistent protection compared to the unvaccinated group but did not induce this lung parenchymal population. It is only with mucosal BCG vaccination that the resident memory CD4^+^ T cells in the lung parenchyma are induced [20], [21], [36], suggesting that systemic BCG has a different mechanism of protection. In a recent study, intravenous BCG also protected against *M.tb* infection in rhesus [40]. The intravenous vaccination induced antigen-responsive CD4^+^ and CD8^+^ T in the lung parenchyma, illustrating different routes might work via different mechanisms [40].

Boosting BCG with C failed to further enhance the memory CD4^+^ T cells in the lung parenchyma of BCG-vaccinated mice. This result could explain the inferior protection of B-C compared to B-C-M vaccination. The importance of memory CD4^+^ T cells in protection is further highlighted with the decrease of this cell population with time post-vaccination in the B-C-M regimen. There are reports to suggest that CD4 T_RM_ cells are not very long-lived [21]. Bull *et al.* showed that the intranasal administration of BCG was more protective compared to the intradermal vaccination at 4 weeks post vaccination [20]. This protection was associated with the frequency of T_RM_, with both decreasing in later time points post vaccination [21] which is consistent with our finding. Taken together, Ag85A-specific lung parenchymal CXCR3^+^ KLRG1^-^ CD4^+^ T cells may associate with protection against *M.tb* infection. As far as effector responses are concerned, our data show that a proportion of these memory cells had an effector phenotype and were able to secrete IFN-γ and TNF-α upon antigen-specific stimulation.

Lung parenchymal Ag85A-specific CD8^+^ T cells with a lung-homing (CXCR3^+^ KLRG1^-^) and resident memory phenotype (CD44^hi^ CD62L^low^ CD69^+^ CD103^+^) were increased in B-C, B-C-Mid and B-C-Min compared to BCG vaccinated mice at 4, 8 and 20 weeks post vaccination. Resident memory CD8^+^ T cells were enhanced after vaccination with BCG and other subunit vaccines [36], [41]. In contrast to the CD4^+^ responses, MVA85A could not boost CD8^+^ responses induced by B-C vaccination. This could be because ChAdOx1.85A is a potent inducer of CD8^+^ T cells [33], [42]. At the 4 and 8 week-time points, these cells were present in all groups, including the B-C group where protection was not observed. At 20 weeks post vaccination, there was a trend for lower CFU in all groups including the B-C group suggesting that perhaps CD8^+^ cells might also contribute as the CD4^+^ numbers were declining. In a recent study, the protective function of resident memory CD8^+^ T cells has been shown using mucosal adoptive transfer of the airway-CD8^+^ T_RM_ cells but only after the recipients had BAL removal before the transfer [36].

Antibodies may have a potential role in protection against *M.tb,* particularly when induced mucosally [22], [26], [27], [43], [44]. For example, intranasal vaccination of *M.tb* antigen-coated bacterial spores or antigen-coated nanoparticles enhanced antigen-specific IgG responses in the sera and IgA responses in the BAL, resulting in a reduction of bacterial CFU following *M.tb* challenge [28], [29]. Intranasal ChAdOx1.85A vaccination induced Ag85A-specific systemic IgG responses, which were maintained after an i.d. or an i.n. MVA85A. An intranasal prime with chimpanzee adenovirus followed by an intramuscular boost with MVA, both expressing RSV antigens, has been shown to increase antigen-specific IgG titre in the serum of CD1 mice compared to mice who received both vaccines systemically [45]. In agreement with this work, intranasal boosting of ChAdOx1.85A with MVA85A seems to be more efficient at maintaining a strong antibody response, compared to intradermal MVA85A boost. However, in our study antibody responses did not directly correlate with the protection, although we can not exclude a contributing role. More recently, a human study showed an important role of antibodies specific to mycobacterial surface protein compared to ones specific to secreted antigens [46]. This study suggests that antigen selection might be important to the protective capacity of antibodies induced by vaccination.

Immune responses which associated with protection afforded by a heterologous prime-boost regimen, were revealed after combining intravascular staining with tetramer and surface staining for resident memory and protective lung-homing phenotypes. This approach specifically identified lung-parenchymal cells and allowed the detailed phenotypic characterization of T cell responses induced by B-C-M vaccination. Adoptive transfer of antigen-specific lung parenchymal CD4^+^ T cells could confirm their protective role. It would also be of interest and importance to analyse the dynamics and role of these cells following *M.tb* infection. as well. The lung parenchymal CD4^+^ T cells with the expression of protective phenotype identified in this study could be a target for new TB vaccine candidates in development.

## 4 Methods

### 4.1 Vaccines

Bacillus Calmette–Guérin (BCG) Pasteur was cultured in-house in 7H9 Broth (BD, UK) with 10% Middlebrook ADC Enrichment and 0.05% Tween-80 until mid-log phase. BCG was spun down and resuspended in PBS before storing at −80 °C.

Replication-deficient chimpanzee adenovirus vector (ChAdOx1) [33], [42] and modified vaccinia Ankara (MVA) [47] expressing antigen Ag85A were constructed in-house by the Vector Core Facility at the Jenner Institute.

### 4.2 Mice

Six to eight-week-old female BALB/c mice were purchased from Envigo (Cambridgeshire, UK). Mice were housed in the Functional Genomics Facility in the Wellcome Trust Centre for Human Genetics, University of Oxford. All animal experiments were approved and licensed by the UK Home Office under license 30/2889 and P9804B4F1.

### 4.3 Immunisations

Immunisations were performed under inhalational anaesthesia using isoflurane (Sigma). BCG was intradermally (i.d.) administered at a dose of 4x10^5^ colony forming units (CFU) at the ears of mice (25 µl on each side). Mice were vaccinated with 5x10^6^ plaque forming units (pfu) of MVA via the i.d or i.n. route. ChAdOx1 was delivered intranasally (i.n.) at a dose of 1x10^8^ infectious units (ifu) in a final volume of 50 µl.

### 4.4 *M.tb* Challenge

Aerosol challenge with *M.tb* was conducted using a Biaera AeroMP®-controlled nebuliser (Biaera technologies, Hagertown, USA) contained in a biosafety level 3 TCOL isolator (Total containment Oxford Ltd.) Mice were loaded in nose-only restainers and exposed to aerosolised bacteria. *M.tb* Erdman *K*01 (TMC107) (BEI resources; Manassas, USA) was prepared at 1x10^6^ CFU/ml and loaded in the nebuliser. The machine was programmed to run for 10 min followed by a 5-minute purge at airflow 10 L/min and pressure 20 lb per square inch gauge (psig). The infectious dose was 50–100 CFU per animal, verified 24 h after the challenge using two mice per experiment.

### 4.5 Colony forming unit (CFU) determination

To measure bacterial CFU in the organs of *M.tb* infected mice, lungs and spleen were harvested into CK28-R tubes containing 2.8 mm ceramic beads (Stretton Scientific, Derbyshire, UK), filled with 1 ml of PBS. The samples were homogenised twice using a Precellys 24 (Stretton Scientific, Derbyshire, UK) for 20 s at 5500 revolutions per minute (rpm). Homogenised specimens were serially diluted in PBS and plated in duplicate on Modified 7H11 agar plates (Animal and Plant Health Agency, UK). The colony count was obtained after a 5-week incubation at 37 °C.

### 4.6 Intravascular staining

BALB/c mice were pre-heated at 37 °C in a heat box for 10 min. 2.5 μg of FITC-conjugated anti-CD45.2 (eBiosciences) was injected intravenously into the tail of the mice in a volume of 100 μl. One minute after the injection, mice were sacrificed, and the lungs were collected. Lungs were placed in a C-tube (Miltenyi Biotec) containing the digestion media (RPM1 with collagenase and DNase). A gentleMACS dissociator was used to homogenise the lungs according to the manufacturer’s protocol. The homogenised lungs were incubated in a shaking incubator at 37 °C for 1 h. The content was poured through a 100 µm cell strainer (Greiner Bio-One) into a 50 ml tube. The samples were spun for 8 min, 300 × *g* at 4 °C. After the spinning, the cells were treated with ACK buffer for 5 min and filtered using a 40 µm cell strainer (Greiner Bio-One) before spinning as above. The cell pellet was resuspended in complete media. To determine total cell numbers, the cells were counted using CASY counter.

### 4.7 Cell surface staining and tetramer staining

2x10^6^ lung cells and spleen cells were stained per sample/stimulation. Cells were washed with PBS and centrifuged at 600 × *g* at 4 °C. To identify antigen-specific CD4^+^ and CD8^+^ T cells, Ag85A-specific tetramer mixture diluted 1:100 in PBS (Class II: I-E(d) *M.tb* Ag85A 165–178 LPGWLQANRHVKPT, Class I: H-2L(d) *M.tb* Ag85B 110–118 MPVGGQSSF) (NIH Tetramer Core Facility, USA) was added for 25 min at 4 °C. Cells were washed twice with PBS and surface staining was performed. Cells were initially stained with live/dead Aqua fixable dye (1:1000 in PBS; Invitrogen) for 10 min followed by 30 min incubation with anti-CD19, CD4, CD44, CD69, CX3CR1, CD103 (BioLegend), anti-CD8a, CD62L, CD11a,KLRG1, CXCR3 (eBioscience) and anti-mouse CD16/CD32 receptor antibody (1:200, eBioscience) diluted in 2% Bovine Serum Albumin (BSA) (Sigma-Aldrich) in PBS (FACS buffer). The stained cells were washed and fixed with 100 μl of 1% Paraformaldehyde, for 10 min at room temperature (RT), spun and resuspended in FACS buffer for running on an LSRII flow cytometer (BD Bioscience). The acquired data were analysed using FlowJo version 10 (Tree Star, USA).

### 4.8 Intracellular staining

To detect cytokine producing T cells, 2x10^6^ lung cells were stimulated with 2 μg/ml of Ag85A 66 peptide pool (15mers overlapping by 10 amino acids) or purified protein derivative of *M.tb* (PPD-T), or complete media as an unstimulated control at 37 °C and 5% CO_2_. After 2 h, GolgiPlug (1:1000, BD Bioscience) was added for further 4 h. The stimulated cells were stored at 4 °C for staining the next day. After the surface staining, the cells were fixed and permeabilised using CytoFix (BD Biosciences) as per the manufacturer’s protocol*.* Cells were stained with anti- IFN-γ, TNF-α and Fc block (eBioscience) in diluted CytoPerm buffer (BD Biosciences) for 1 h at 4 °C. Cells were washed with CytoPerm buffer and resuspended in FACS buffer. All samples were run and analysed as above.

### 4.9 Enzyme-linked immunosorbent assay (ELISA)

Enzyme-linked immunosorbent assay (ELISA) was used to measure Ag85A and PPD specific antibodies in BAL fluid and serum. Maxisorp 96-well plates (ThermoFisher Scientific) were coated with diluted recombinant 85A (r85A) (2 µg/ml for detecting IgG, 10 µg/ml for detecting IgA) or PPD-T (6.67 µg/ml) in PBS incubated overnight at RT. The plates were washed 6 times with PBS/Tween-20 (Sigma-Aldrich) and blocked with 2.5% BSA (Sigma-Aldrich) in PBS for 1 h at RT. After blocking, the plates were washed with the same method. Serum samples were prepared at an initial dilution of 1:50 whereas BAL samples were plated neat. Serial dilutions were done in 0.1% BSA/PBS/Tween or PBS/Tween for serum and BAL respectively. Fifty µl of the samples were incubated for 2 h at RT. After washing with PBS/Tween-20, alkaline phosphatase conjugated anti-IgG (1:5000) or anti-IgA (1:1000) (Sigma-Aldrich) diluted in PBS/Tween was added and incubated for 1 h at RT. The plates were washed before addition of development buffer (1 mg/ml of 4-nitrophenylphosphate tablet (Sigma-Aldrich)) diluted in diethanolamine buffer (ThermoFisher Scientific). The reaction was developed at RT and optical density (OD) was read at 405 nm (Gen5 software) using a spectrophotometer (BioTeK Microplate Reader). Mean optical density of duplicate blank wells were subtracted from mean OD of test samples to obtain adjusted OD. Serum from naïve mice was used as negative control. Previously determined high responders were used as positive control.

### 4.10 Statistical analysis

Statistical analyses were performed using GraphPad Prism 7 software (GraphPad Software Inc.). To determine the statistical significance, Mann Whitney test was used to compare two groups while, Kruskal-Wallis followed by Dunn's multiple comparisons test was performed when analysing multiple groups. Values of p ≤ 0.05 were considered as statistically significant. *p ≤ 0.05, **p ≤ 0.01, ***p ≤ 0.001, ****p ≤ 0.0001.

## Author contributions

NP, ES, NB, JP, RHK performed the experiments. NP, ES and HMcS designed experiments and interpreted the data. NP wrote the manuscript with input from ES and HMcS. All authors had access to the data and approved the manuscript before it was submitted.

## Funding

This research was funded in whole, or in part, by the Wellcome Trust. HMcS is a Wellcome Trust Investigator (grant code WT 206331/Z/17/Z). For the purpose of open access, the author has applied a CC BY public copyright license to any Author Accepted Manuscript version arising from this submission.

This project received funding from the European Commission Horizon 2020 research and innovation programme under grant agreement No 643,381 (H2020TBVAC). The funders had no role in study design, data collection and analysis, decision to publish, or preparation of the manuscript.

## Declaration of Competing Interest

The authors declare that they have no known competing financial interests or personal relationships that could have appeared to influence the work reported in this paper.

## References

[1] Shameer A. (2019). WHO. Global tuberculosis report.

[2] Rodrigues L.C., Diwan V.K., Wheeler J.G. (1993). Protective effect of BCG against tuberculous meningitis and miliary tuberculosis: a meta-analysis. Int J Epidemiol..

[3] Geldmacher C., Ngwenyama N., Schuetz A., Petrovas C., Reither K., Heeregrave E.J. (2010). Preferential infection and depletion of Mycobacterium tuberculosis-specific CD4 T cells after HIV-1 infection. J Exp Med.

[4] Geldmacher C., Schuetz A., Ngwenyama N., Casazza J.P., Sanga E., Saathoff E. (2008). Early depletion of Mycobacterium tuberculosis-specific T helper 1 cell responses after HIV-1 infection. J Infect Dis..

[5] Geldmacher C., Zumla A., Hoelscher M. (2012). Interaction between HIV and Mycobacterium tuberculosis: HIV-1-induced CD4 T-cell depletion and the development of active tuberculosis. Curr Opin HIV AIDS..

[6] Donovan M.L., Schultz T.E., Duke T.J., Blumenthal A. (2017). Type I Interferons in the Pathogenesis of Tuberculosis: Molecular Drivers and Immunological Consequences. Front Immunol..

[7] Caccamo N., Meraviglia S., La Mendola C., Guggino G., Dieli F., Salerno A. (2006). Phenotypical and functional analysis of memory and effector human CD8 T cells specific for mycobacterial antigens. J Immunol..

[8] Stenger S., Hanson D.A., Teitelbaum R., Dewan P., Niazi K.R., Froelich C.J. (1998). An antimicrobial activity of cytolytic T cells mediated by granulysin. Science.

[9] Chen C.Y., Huang D., Wang R.C., Shen L., Zeng G., Yao S. (2009). A critical role for CD8 T cells in a nonhuman primate model of tuberculosis. PLoS Pathog..

[10] Fletcher H.A., Snowden M.A., Landry B., Rida W., Satti I., Harris S.A. (2016). T-cell activation is an immune correlate of risk in BCG vaccinated infants. Nat Commun..

[11] Kaech S.M., Wherry E.J., Ahmed R. (2002). Effector and memory T-cell differentiation: implications for vaccine development. Nat Rev Immunol..

[12] Henao-Tamayo M.I., Ordway D.J., Irwin S.M., Shang S., Shanley C., Orme I.M. (2010). Phenotypic definition of effector and memory T-lymphocyte subsets in mice chronically infected with Mycobacterium tuberculosis. Clin Vaccine Immunol..

[13] Mackay L.K., Braun A., Macleod B.L., Collins N., Tebartz C., Bedoui S. (2015). Cutting edge: CD69 interference with sphingosine-1-phosphate receptor function regulates peripheral T cell retention. J Immunol..

[14] Teijaro J.R., Turner D., Pham Q., Wherry E.J., Lefrancois L., Farber D.L. (2011). Cutting edge: Tissue-retentive lung memory CD4 T cells mediate optimal protection to respiratory virus infection. J Immunol..

[15] Turner D.L., Bickham K.L., Thome J.J., Kim C.Y., D'Ovidio F., Wherry E.J. (2014). Lung niches for the generation and maintenance of tissue-resident memory T cells. Mucosal Immunol..

[16] Anderson K.G., Mayer-Barber K., Sung H., Beura L., James B.R., Taylor J.J. (2014). Intravascular staining for discrimination of vascular and tissue leukocytes. Nat Protoc..

[17] Sakai S., Kauffman K.D., Schenkel J.M., McBerry C.C., Mayer-Barber K.D., Masopust D. (2014). Cutting edge: control of Mycobacterium tuberculosis infection by a subset of lung parenchyma-homing CD4 T cells. J Immunol..

[18] Shin H., Iwasaki A. (2012). A vaccine strategy that protects against genital herpes by establishing local memory T cells. Nature.

[19] Woodworth J.S., Cohen S.B., Moguche A.O., Plumlee C.R., Agger E.M., Urdahl K.B. (2017). Subunit vaccine H56/CAF01 induces a population of circulating CD4 T cells that traffic into the Mycobacterium tuberculosis-infected lung. Mucosal Immunol..

[20] Bull N.C., Stylianou E., Kaveh D.A., Pinpathomrat N., Pasricha J., Harrington-Kandt R. (2018). Enhanced protection conferred by mucosal BCG vaccination associates with presence of antigen-specific lung tissue-resident PD-1(+) KLRG1(-) CD4(+) T cells. Mucosal Immunol.

[21] Bull N.C., Kaveh D.A., Garcia-Pelayo M.C., Stylianou E., McShane H., Hogarth P.J. (2018). Induction and maintenance of a phenotypically heterogeneous lung tissue-resident CD4(+) T cell population following BCG immunisation. Vaccine.

[22] Orme I.M. (2016). Vaccines to prevent tuberculosis infection rather than disease: Physiological and immunological aspects. Tuberculosis..

[23] Glatman-Freedman A., Casadevall A. (1998). Serum therapy for tuberculosis revisited: reappraisal of the role of antibody-mediated immunity against Mycobacterium tuberculosis. Clin Microbiol Rev..

[24] Hussain R., Shiratsuchi H., Ellner J.J., Wallis R.S. (2000). PPD-specific IgG1 antibody subclass upregulate tumour necrosis factor expression in PPD-stimulated monocytes: possible link with disease pathogenesis in tuberculosis. Clin Exp Immunol..

[25] Rodriguez A., Tjarnlund A., Ivanji J., Singh M., Garcia I., Williams A. (2005). Role of IgA in the defense against respiratory infections IgA deficient mice exhibited increased susceptibility to intranasal infection with Mycobacterium bovis BCG. Vaccine..

[26] White A.D., Sarfas C., West K., Sibley L.S., Wareham A.S., Clark S. (2015). Evaluation of the Immunogenicity of Mycobacterium bovis BCG Delivered by Aerosol to the Lungs of Macaques. Clin Vaccine Immunol..

[27] Khera A.K., Afkhami S., Lai R., Jeyanathan M., Zganiacz A., Mandur T. (2015). Role of B Cells in Mucosal Vaccine-Induced Protective CD8+ T Cell Immunity against Pulmonary Tuberculosis. J Immunol..

[28] Reljic R., Sibley L., Huang J.M., Pepponi I., Hoppe A., Hong H.A. (2013). Mucosal Vaccination against Tuberculosis Using Inert Bioparticles. Infect Immun.

[29] Stylianou E., Diogo G.R., Pepponi I., van Dolleweerd C., Arias M.A., Locht C. (2014). Mucosal delivery of antigen-coated nanoparticles to lungs confers protective immunity against tuberculosis infection in mice. Eur J Immunol.

[30] McShane H., Pathan A.A., Sander C.R., Keating S.M., Gilbert S.C., Huygen K. (2004). Recombinant modified vaccinia virus Ankara expressing antigen 85A boosts BCG-primed and naturally acquired antimycobacterial immunity in humans. Nat Med..

[31] Scriba T.J., Tameris M., Mansoor N., Smit E., van der Merwe L., Mauff K. (2011). Dose-finding study of the novel tuberculosis vaccine, MVA85A, in healthy BCG-vaccinated infants. J Infect Dis..

[32] Tameris M.D., Hatherill M., Landry B.S., Scriba T.J., Snowden M.A., Lockhart S. (2013). Safety and efficacy of MVA85A, a new tuberculosis vaccine, in infants previously vaccinated with BCG: a randomised, placebo-controlled phase 2b trial. Lancet.

[33] Dicks M.D., Spencer A.J., Edwards N.J., Wadell G., Bojang K., Gilbert S.C. (2012). A novel chimpanzee adenovirus vector with low human seroprevalence: improved systems for vector derivation and comparative immunogenicity. PLoS ONE.

[34] Stylianou E., Griffiths K.L., Poyntz H.C., Harrington-Kandt R., Dicks M.D., Stockdale L. (2015). Improvement of BCG protective efficacy with a novel chimpanzee adenovirus and a modified vaccinia Ankara virus both expressing Ag85A. Vaccine..

[35] Anderson K.G., Sung H., Skon C.N., Lefrancois L., Deisinger A., Vezys V. (2012). Cutting edge: intravascular staining redefines lung CD8 T cell responses. J Immunol..

[36] Perdomo C., Zedler U., Kuhl A.A., Lozza L., Saikali P., Sander L.E. (2016). Mucosal BCG Vaccination Induces Protective Lung-Resident Memory T Cell Populations against Tuberculosis. MBio..

[37] Jeyanathan M., Afkhami S., Khera A., Mandur T., Damjanovic D., Yao Y. (2017). CXCR3 Signaling Is Required for Restricted Homing of Parenteral Tuberculosis Vaccine-Induced T Cells to Both the Lung Parenchyma and Airway. J Immunol..

[38] Wang J., Thorson L., Stokes R.W., Santosuosso M., Huygen K., Zganiacz A. (2004). Single mucosal, but not parenteral, immunization with recombinant adenoviral-based vaccine provides potent protection from pulmonary tuberculosis. J Immunol..

[39] Jeyanathan M., Shao Z., Yu X., Harkness R., Jiang R., Li J. (2015). AdHu5Ag85A Respiratory Mucosal Boost Immunization Enhances Protection against Pulmonary Tuberculosis in BCG-Primed Non-Human Primates. PLoS ONE.

[40] Darrah P.A., Zeppa J.J., Maiello P., Hackney J.A., Wadsworth M.H., Hughes T.K. (2020). Prevention of tuberculosis in macaques after intravenous BCG immunization. Nature.

[41] Stylianou E., Harrington-Kandt R., Beglov J., Bull N., Pinpathomrat N., Swarbrick G.M. (2018). Identification and Evaluation of Novel Protective Antigens for the Development of a Candidate TB Subunit Vaccine. Infect Immun.

[42] Dicks M.D., Guzman E., Spencer A.J., Gilbert S.C., Charleston B., Hill A.V. (2015). The relative magnitude of transgene-specific adaptive immune responses induced by human and chimpanzee adenovirus vectors differs between laboratory animals and a target species. Vaccine..

[43] Prados-Rosales R., Carreno L.J., Batista-Gonzalez A., Baena A., Venkataswamy M.M., Xu J. (2014). Mycobacterial membrane vesicles administered systemically in mice induce a protective immune response to surface compartments of Mycobacterium tuberculosis. MBio..

[44] Hamasur B., Haile M., Pawlowski A., Schroder U., Williams A., Hatch G. (2003). Mycobacterium tuberculosis arabinomannan-protein conjugates protect against tuberculosis. Vaccine..

[45] Pierantoni A., Esposito M.L., Ammendola V., Napolitano F., Grazioli F., Abbate A. (2015). Mucosal delivery of a vectored RSV vaccine is safe and elicits protective immunity in rodents and nonhuman primates. Molecular therapy Methods & clinical development..

[46] Li H., Wang X.X., Wang B., Fu L., Liu G., Lu Y. (2017). Latently and uninfected healthcare workers exposed to TB make protective antibodies against Mycobacterium tuberculosis. Proc Natl Acad Sci U S A..

[47] McShane H., Brookes R., Gilbert S.C., Hill A.V. (2001). Enhanced immunogenicity of CD4(+) t-cell responses and protective efficacy of a DNA-modified vaccinia virus Ankara prime-boost vaccination regimen for murine tuberculosis. Infect Immun..

