# Supplementary material for: Using an effective TB vaccination regimen to identify immune responses associated with protection in the murine model
Source: Vaccine. 2021 Mar 1;39(9):1452–62. doi: 10.1016/j.vaccine.2021.01.034 (PMC7903242; doi:10.1016/j.vaccine.2021.01.034)
Supplement: Supplementary data 1 [file mmc1.docx]

**Supplementary data**


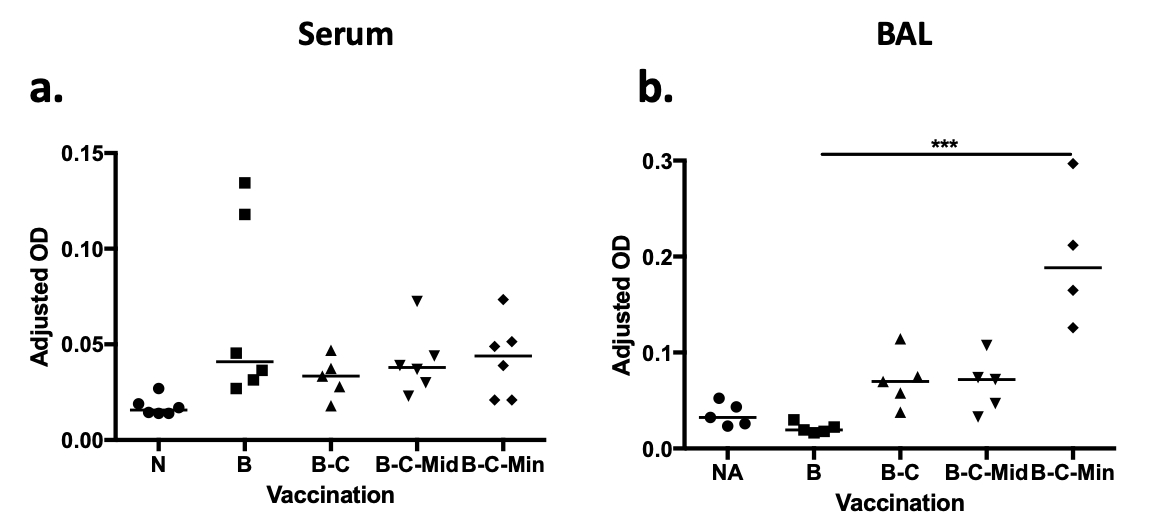


**Supplement 1. PPD-specific antibody responses in the circulation and airways of immunised and control mice at 4 weeks post vaccination.** Mice were vaccinated as shown in FIG 2a. (a) Serum and (b) BAL samples were analyzed using ELISA. (a) PPD-specific total IgG at 1:450 dilution in serum were presented. (b) PPD-specific IgA were measured in undiluted BAL samples. Each symbol represents one animal and the line is the median of each group (n=4-6 mice). Data are representative of two independent experiments. Statistical significance was determined using Kruskal-Wallis test with Dunn's multiple comparisons test. ***, p≤0.001.

**
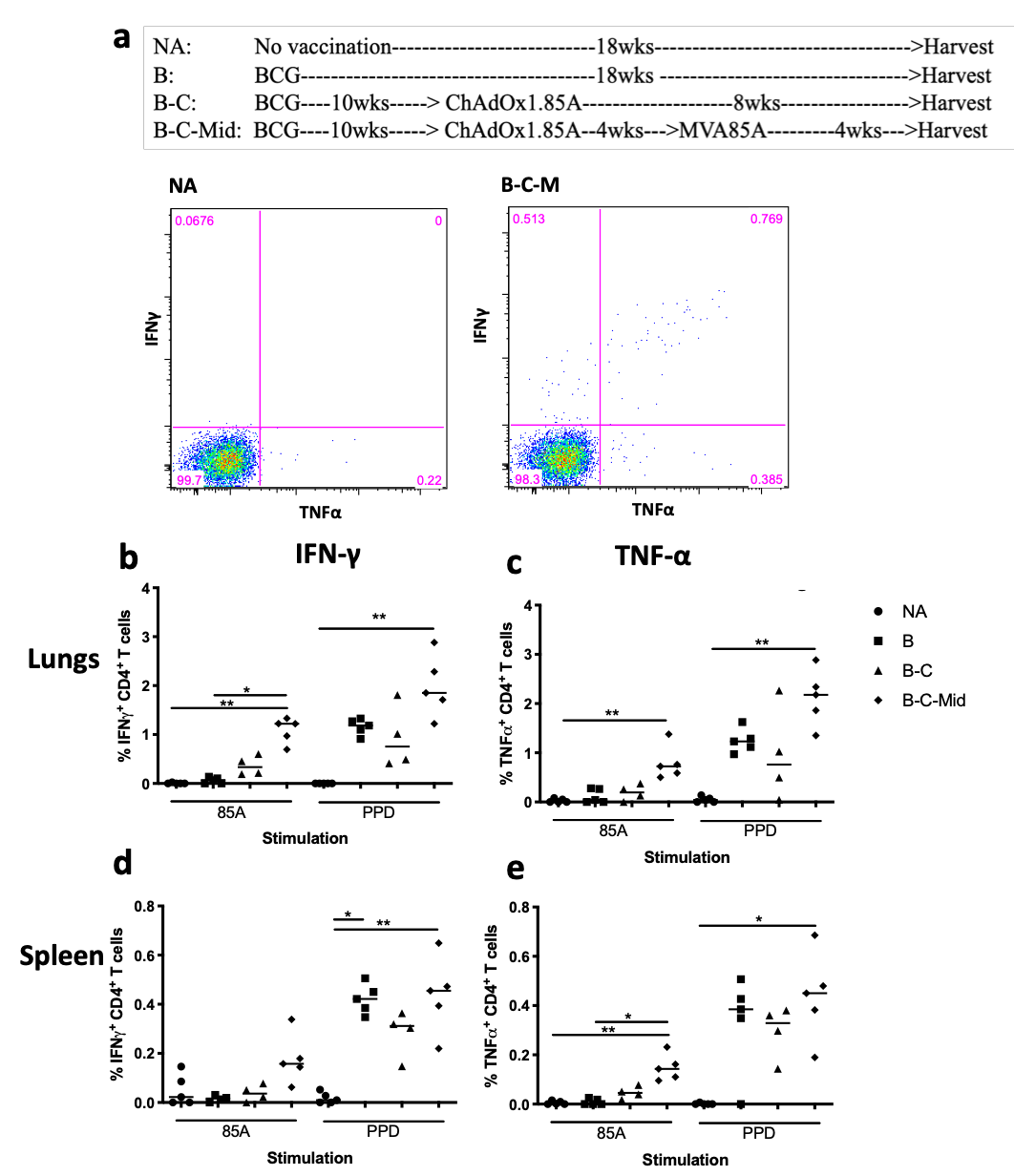
**

**Supplement 2. CD4^+^ T cell cytokine responses in the lungs and spleen of naïve and vaccinated mice.** (a) Experimental schema. Murine samples were harvested at 4 weeks after the last vaccination in B-C-Mid, at 8 weeks post ChAdOx1 vaccination in B-C group. Lung and spleens cells were stimulated *ex-vivo* with Ag85A peptide pool (85A) and purified protein derivative from *M.tb* (PPD). Representative flow plots show cytokine-producing CD4^+^ T cells in the lungs of naïve (NA) (Left panel) and B-C-M (Right panel) group. (b) Percentage of IFN-γ^+^ and (c) TNF-α^+^ CD4^+^ T cells in lungs and spleens (d) and (e) respectively. The experiment was repeated twice with similar results. Each symbol represents one animal and the line is the median of each group (n=4-5 mice). Statistical significance was determined using Kruskal-Wallis test with Dunn's multiple comparisons test. *p≤0.05; **p≤0.01.

**
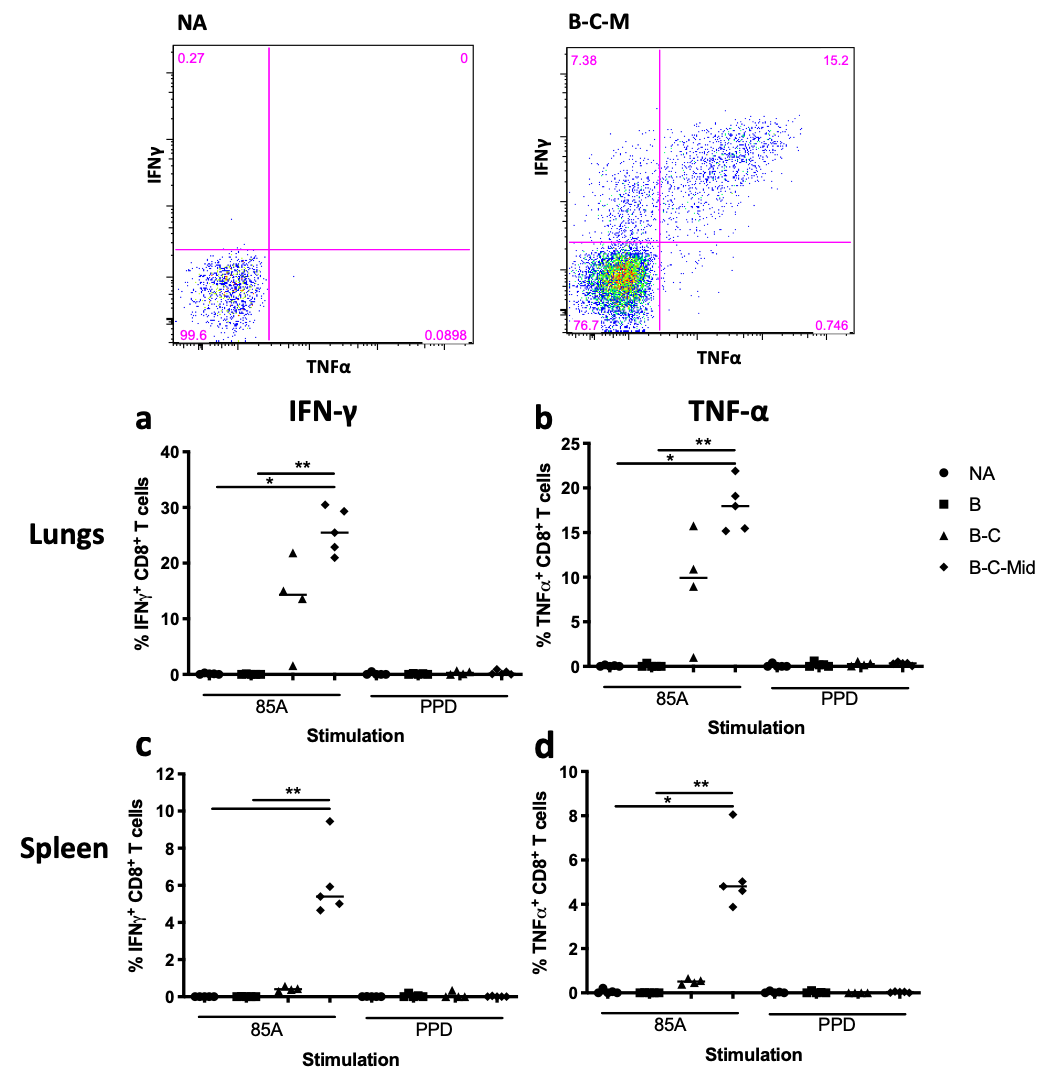
**

**Supplement 3. CD8^+^ T cell cytokine responses in lungs and spleen of naïve and vaccinated mice.** Representative flow plots show cytokine-producing CD8^+^ T cells in the lungs of naïve (NA) (Left panel) and B-C-M (Right panel) groups. (a) Percentage of IFN-γ^+^ and (b) TNF-α^+^ CD8^+^ T cells in lungs and spleens (c) and (d) respectively. The experiment was repeated twice with similar results. Each symbol represents one animal and the line is the median of each group (n=4-5 mice). Statistical significance was determined using Kruskal-Wallis test with Dunn's multiple comparisons test. *, p≤0.05; **, p≤0.01.

**Appendix**


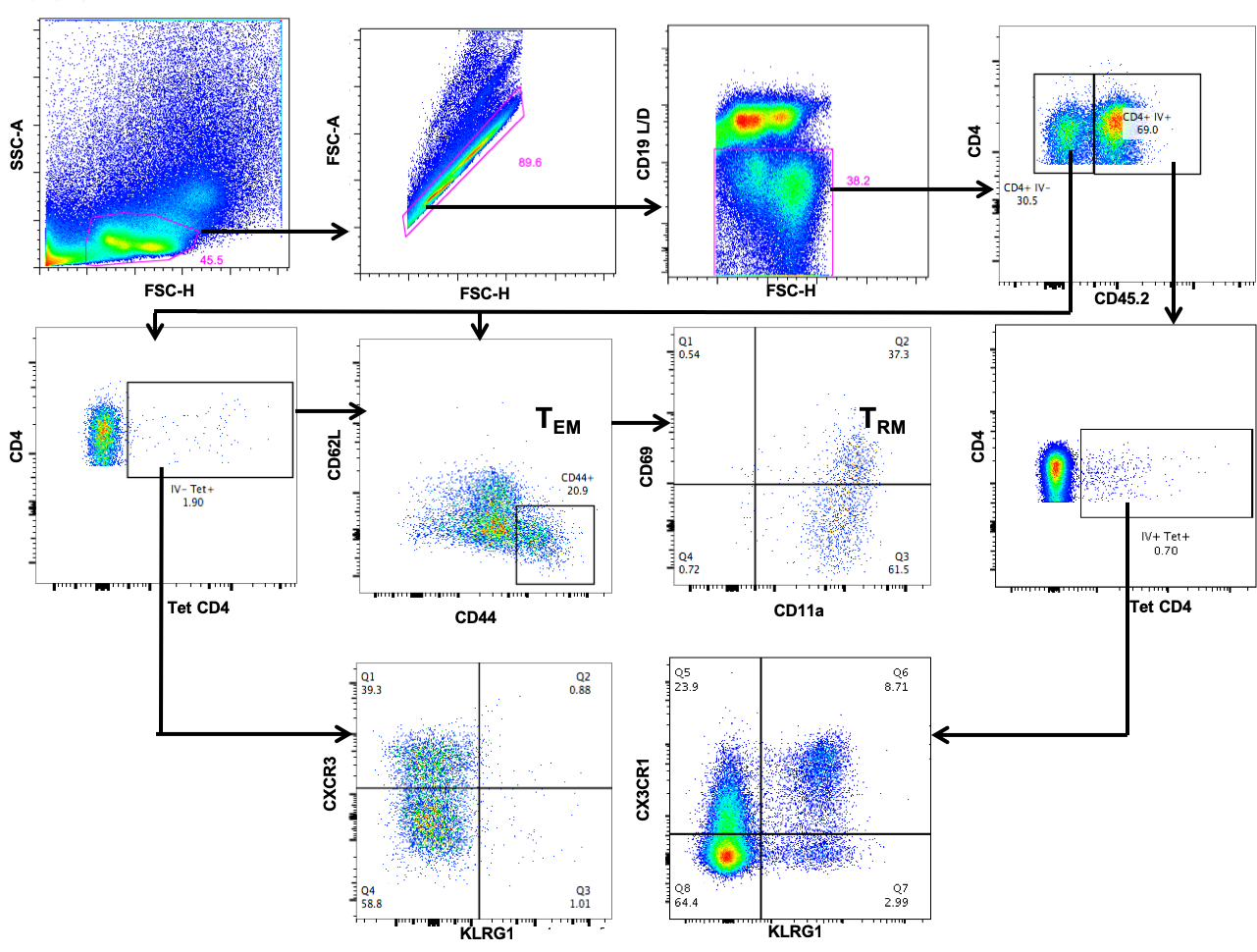


**Appendix 1. Gating strategy for the identification of lung-parenchymal CD4^+^ T cells expressing resident memory and lung-homing markers.**


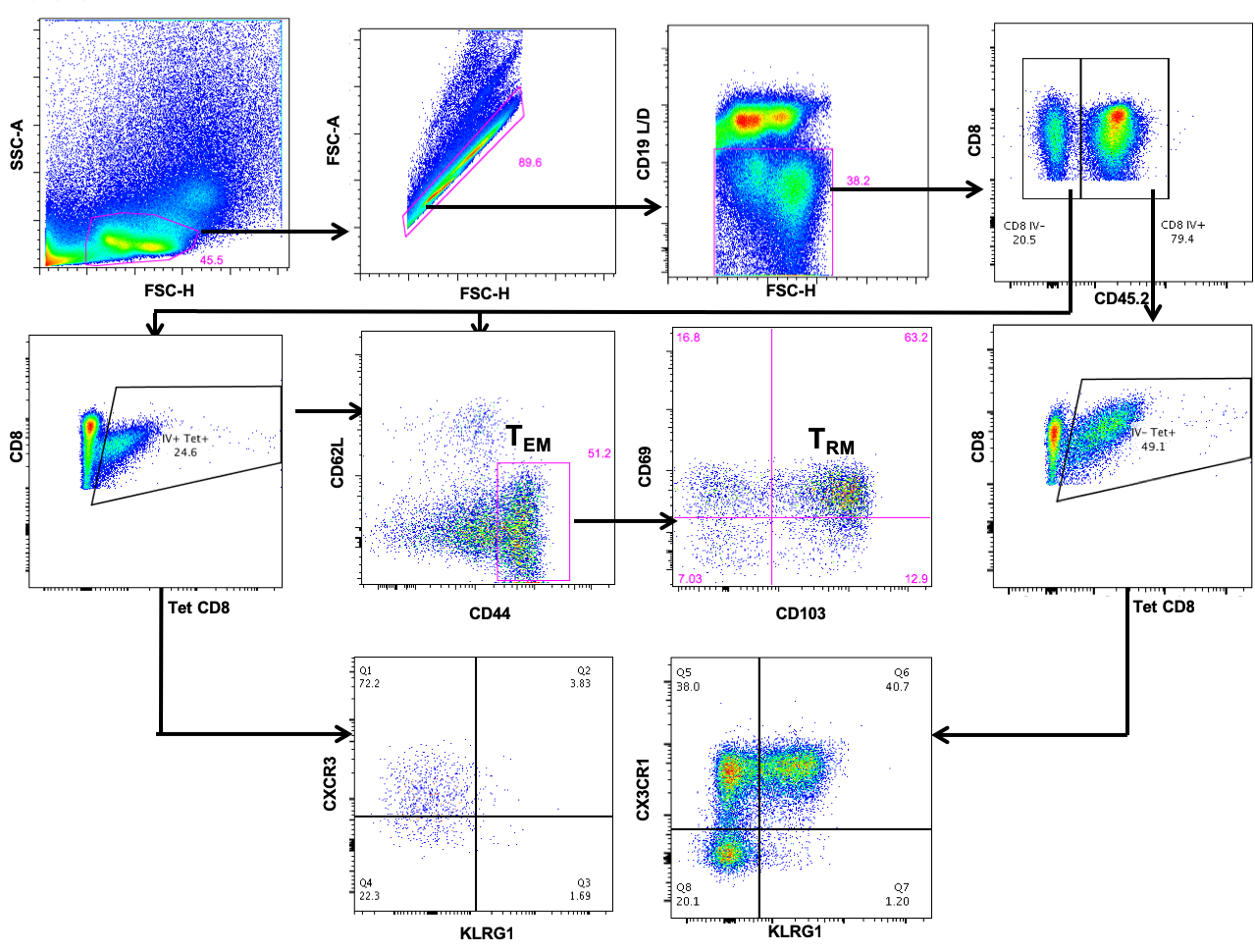


**Appendix 2. Gating strategy for the identification of lung-parenchymal CD8^+^ T cells expressing resident memory and lung-homing markers.**

**
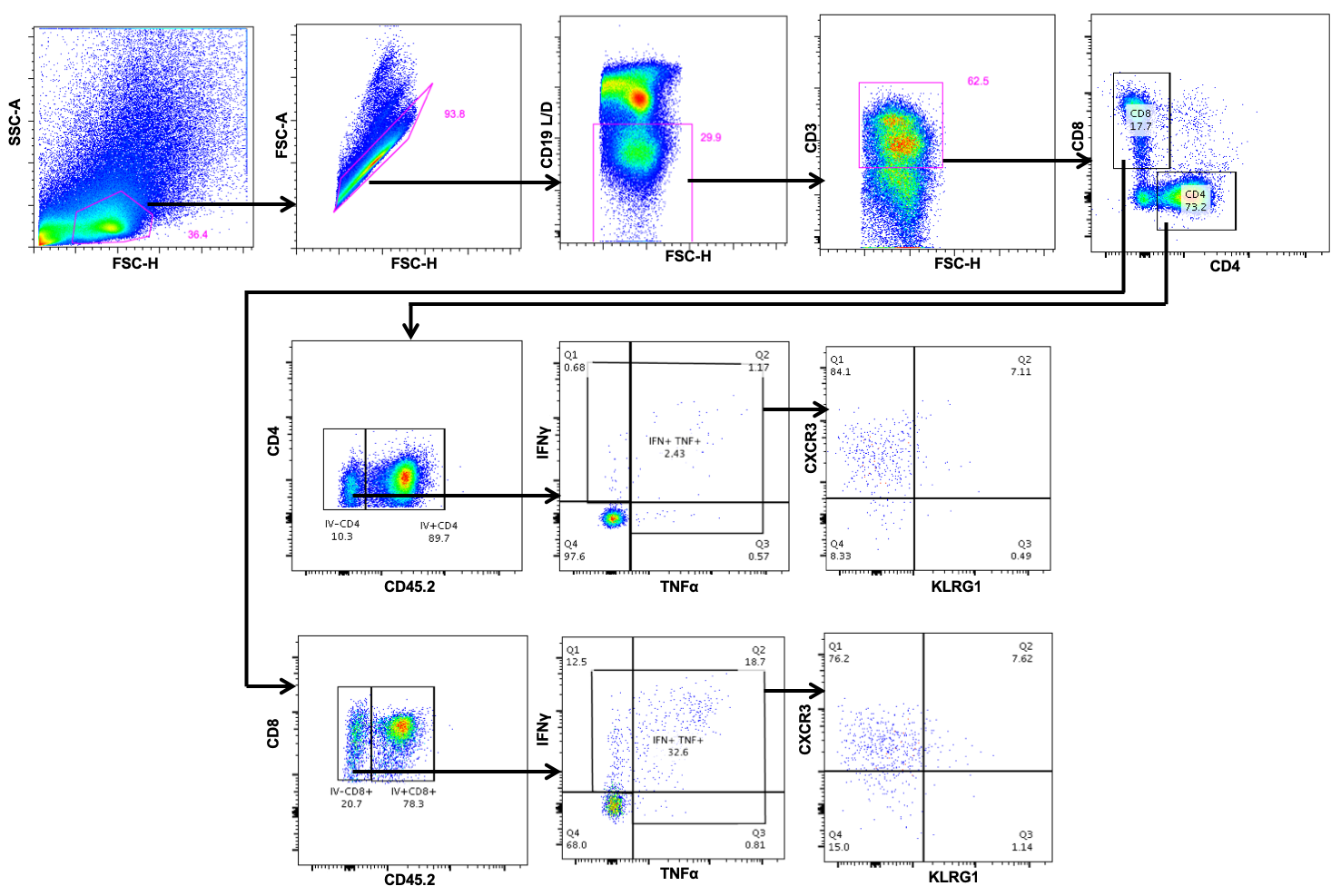
**

**Appendix 3. Gating strategy for the identification of cytokine producing lung-parenchymal T cells expressing lung-homing markers.**
